# Severe neuromuscular immune-related adverse events of immune checkpoint inhibitors at national cancer center in Korea

**DOI:** 10.1007/s00432-022-04516-x

**Published:** 2022-12-10

**Authors:** Jae-Won Hyun, Ki Hoon Kim, Su-Hyun Kim, Ho Jin Kim

**Affiliations:** Department of Neurology, Research Institute and Hospital of National Cancer Center, 323 Ilsan-Ro, Ilsandong-gu, Goyang, Korea

**Keywords:** Immune checkpoint inhibitor, Cancer, Neuromuscular, Adverse event

## Abstract

**Purpose:**

Neuromuscular immune-related adverse events (irAEs) associated with immune checkpoint inhibitors (ICIs) have been increasingly recognized as a consequence of expanding use of ICIs in advanced cancers. We aimed to evaluate the frequency, phenotypes, rescue treatment, and clinical outcomes of severe neuromuscular irAEs of ICIs at National Cancer Center (NCC), Korea.

**Materials and methods:**

Consecutive patients with newly developed severe neuromuscular irAEs (common terminology criteria for adverse events grade 3 or greater) after ICI treatment at NCC in Korea between December 2018 and April 2022 were included by searching neuromuscular diagnostic codes in electronic medical records and/or reviewing neurological consultation documentations.

**Results:**

Of the 1,503 ICI-treated patients, nine (0.6%) experienced severe neuromuscular irAEs; five with pembrolizumab and four with atezolizumab. The patients included five women and four men; their median age at onset was 59 years. The irAEs included Guillain–Barre syndrome (*n* = 5) and myasthenia gravis (MG) crisis with myositis (*n* = 4), and developed after a median of one (range 1–5) ICI cycle. The median modified Rankin score (mRS) was 4 (range 3–5) at the nadir. ICIs were discontinued in all patients, and rescue immunotherapy included corticosteroids (*n* = 9), intravenous immunoglobulin (*n* = 7), and plasmapheresis (*n* = 2). Eight patients showed improvements, with a median mRS of 3 (range 1–4); however, one patient (who had MG crisis with myocarditis) died.

**Conclusions:**

In this real-world monocentric study, ICI-induced neuromuscular irAEs were rare but potentially devastating; thus, physicians should remain vigilant to enable prompt recognition and management of irAEs.

## Introduction

Recently, immune checkpoint inhibitors (ICIs) have been increasingly prescribed for treating various tumors, with favorable results, even in patients with advanced cancer (Hoos 2016). In addition to promoting anti-tumor immunity, ICIs can cause immune-related adverse events (irAEs) by breaking immune tolerance (Chen and Mellman 2013). ICI-induced irAEs involve diverse organs, including the neuromuscular system, and neuromuscular irAE phenotypes include Guillain–Barre Syndrome (GBS), myasthenia gravis (MG), and myopathy (Kao et al. 2017; Mancone et al. 2018; Moreira et al. 2019; Dubey et al. 2020; Sechi et al. 2020). ICI-induced severe neuromuscular irAEs are rare but potentially fatal or result in long-term neurological deficits (Kao et al. 2017; Mancone et al. 2018; Moreira et al. 2019; Dubey et al. 2020; Sechi et al. 2020). A high degree of vigilance for these neuromuscular irAEs is required, and early detection and timely treatment are crucial to reduce neurological disability. In this study, we aimed to investigate real-world experiences of ICI-induced severe neuromuscular irAEs at the National Cancer Center (NCC) in Korea to comprehensively characterize their phenotypic spectrum and report clinical outcomes.

## Methods

Patients treated with ICIs, including programmed cell death protein 1 (PD1) (nivolumab, pembrolizumab), programmed death-ligand 1 (PD-L1) (atezolizumab), and cytotoxic T lymphocyte-associated antigen-4 (CTLA-4) (ipilimumab) inhibitors, at the NCC in Korea from December 2018 to April 2022 were screened for documentation of newly developed neuromuscular international classification of diseases (ICD) diagnoses after ICI treatment. Patients with ICD codes relevant to GBS, inflammatory polyneuropathy, MG, and myopathy were included in the study. Additionally, patients referred for neurological consultation and those who experienced a newly developed neuromuscular condition or aggravated MG after ICI treatment were also reviewed for inclusion. Three participants who experienced MG crisis combined with newly developed myositis after ICI treatment were also included, although they had a history of MG (restricted to ocular symptoms and well controlled by symptomatic medication) before initiation of ICI therapy. Patients whose neuromuscular condition was solely attributable to the progression of their primary cancer, other concurrent chemotherapy, or metabolic conditions were excluded. The association between ICI use and neuromuscular conditions was evaluated using the Naranjo scale (Naranjo et al. 1981); the score was calculated as 6 or 7, suggesting a probable association between ICIs and neuromuscular irAEs in the current study.

We focused on Common Terminology Criteria for Adverse Events (CTCAE) grade 3–5 events, because grade 1–2 events are usually asymptomatic and incidentally discovered, and do not require modification of ICI therapy (Puzanov et al. 2017; Haanen et al. 2017). Clinical, electrodiagnostic, and laboratory information were comprehensively reviewed. Using the modified Rankin Scale (mRS) score, the degree of dependence on daily activities (ranging from 0 to 6) was evaluated (Banks and Marotta 2007). Electrodiagnostic tests were performed using the standard methods of the electrodiagnostic laboratory at NCC. This study was approved by the Institutional Review Board of NCC (no. NCC 2022–0142), and the requirement for patient informed consent was waived due to the use of anonymized data.

## Results

In total, data from 1,503 patients treated with ICIs were screened, and seven candidates were comprehensively reviewed; however, one was excluded, because the symptom onset of their neuromuscular condition (GBS) was too far from the administration of ICI therapy (2 years after the start ICI therapy and 78 days after the most recent ICI dose). In addition, through a review of neurological consultations, three patients with MG crisis accompanied by newly developed myositis after ICI administration were also included. These patients had a history of benign ocular MG prior to the first ICI dose. The origin of the primary tumor among the 1,503 patients was lung (*n* = 676), gastrointestinal (*n* = 368), obstetric gynecologic (*n* = 183), and genitourinary (*n* = 114) systems, skin & bone (*n* = 73), head & neck (*n* = 58), breast (*n* = 17), thymus (*n* = 10), and hematological system (*n* = 4).

Nine (0.6%) participants with ICI-induced severe neuromuscular irAEs were ultimately included. The demographic and clinical information of the patients is summarized in Table 1.

**Table 1:** The demographic and clinical information of the patients

Case	Sex/age	Type of cancer	Immune checkpoint inhibitor (ICI)	ICI cycles to onset of neuromuscular toxicity	Days after most recent ICI dose to onset	Clinical phenotype	Clinical symptoms	Electrodiagnostic findings	MRI findings	CSF study	Serum CK	Anti-Ach receptor antibody (normal: 0–0.5)	Concurrent systemic toxicities	CTCAE Grade	mRS-pre	Days after onset to rescue	Rescue treatment for neuromuscular toxicity	mRS- post 2 Mo
1	M/64	NSCLC (adeno)	Atezolizumab	1	24	GBS variant (AMSAN)	Quadriparesis, hypesthesia, dyspnea	Sensorimotor axonal polyradiculoneuropathy	Spine: Cauda equina enhancement	W:7 P:59	Nl	NA	None	G4	5 (vent)	2	IVIG (2 g/kg) + IVMP (1 g/d* 3d)	4
2	F/65	Bladder ca. (transitional)	Atezolizumab	1	16	GBS variant (AMSAN)	Quadriparesis, hypesthesia	Sensorimotor axonal polyradiculoneuropathy	Spine: Cauda equina enhancement	W:41 P:76	Nl	NA	None	G3	4	1	IVIG (2 g/kg) + IVMP (1 g/d* 5d) + PLPH (#5)	3
3	M/58	Prostate ca. (adeno)	Pembrolizumab	5	24	GBS (AIDP)	Paraparesis, hypesthesia	Sensorimotor demyelinating polyradiculoneuropathy	N/A	N/A	Nl	NA	Thyroiditis	G3	4	18	IVIG (2 g/kg) + PD (2 mg/kg)	1
4	F/57	NSCLC (adeno)	Atezolizumab	1	11	GBS variant	Paraparesis, hypesthesia	Sensorimotor polyneuropathy with mixed features (EMG n.d.)	N/S	W:3 P:64	Nl	NA	None	G3	4	20	IVIG (2 g/kg) + PD (1 mg/kg)	3
5	M/61	Tracheal ca. (squamous)	Atezolizumab	1	18	GBS variant	Paraparesis, Hypesthesia, sensory ataxia	Sensorimotor polyneuropathy with mixed features (EMG n.d.)	Spine: Cauda equina enhancement	W:28 P:150	Nl	NA	Thyroiditis	G3	3	4	PD (1 mg/kg)	1
6	F/55	Thymoma (type B2)	Pembrolizumab	1	15	MG crisis + myositis	Ptosis, quadriparesis, dyspnea	N/A	N/A	N/A	56 times ULN	3.44 nmol/L (7.02)*	Myocarditis, hepatitis	G5	5 (vent)	3	IVMP (1 g/d* 3d)	6
7	F/54	Thymoma (type B2)	Pembrolizumab	1	19	MG crisis + myositis	Ptosis, quadriparesis, dyspnea	RNST: post synaptic NMJ disorder	N/A	N/A	2.2 time ULN	6.76 nmol/L (3.70)*	Hepatitis	G4	5 (vent)	1	IVMP (1 g/d* 5d) + IVIG (2 g/kg) + PLPH (#6)	4
8	M/59	Thymoma (type B2)	Pembrolizumab	1	26	MG crisis + myositis	Ptosis, quadriparesis, dyspnea	N/A	N/A	N/A	7.3 times ULN	5.51 nmol/L (4.45)*	Hepatitis	G4	5 (vent)	1	IVMP (1 g/d* 5d) + IVIG (2 g/kg)	4
9	F/64	Thymoma (type B3)	Pembrolizumab	1	12	MG crisis + myositis	Ptosis, quadriparesis, dyspnea	RNST: post synaptic NMJ disorder	N/A	N/A	14.3 times ULN	2.68 nmol/L	Myocarditis, hepatitis	G4	4	6	IVMP (1 g/d* 5d) + IVIG (2 g/kg)	1

*CK* creatine kinase, *Ach* acetylcholine, *CTCAE* Common Terminology Criteria for Adverse Events, *mRS* modified Rankin score, *Mo* month, *M* male, *F* female, *NSCLC* non-small cell lung cancer, *ca* cancer, *GBS* Guillain–Barre Syndrome, *AMSAN* acute motor and sensory axonal neuropathy, *AIDP* acute inflammatory demyelinating polyneuropathy, *MG* myasthenia gravis, *RNST* repetitive nerve stimulation test, *n d* not done, *N/A* not applicable, *NMJ* neuromuscular junction, *Nl* within normal range, *ULN* upper limit normal, (level)* anti-acethylcholine receptor antibody level at the previous benign ocular MG status, *vent* ventilator care, *IVIG* intravenous immunoglobulin, *IVMP* intravenous methylprednisolone, *d* days, *PLPH* plasmapheresis, *PD* prednisone

### Demographics, oncologic characteristics, and ICI treatment

The included participants were four men and five women, and the median age at symptom onset of neuromuscular irAEs was 59 (range 54–65) years. Thymoma was the most common primary tumor (*n* = 4), followed by lung adenocarcinoma (*n* = 2), tracheal squamous cell carcinoma (*n* = 1), bladder transitional cell carcinoma (*n* = 1), and prostate adenocarcinoma (*n* = 1). Excluding the three participants with thymoma who had ocular MG prior to ICI treatment, none of the patients had a history of neuromuscular or autoimmune disease. Neuromuscular irAEs occurred after pembrolizumab (*n* = 5) and atezolizumab (*n* = 4) therapy.

### Neuromuscular manifestations

The most common severe neuromuscular irAE was GBS (*n* = 5), followed by MG crisis with myositis (*n* = 4). Neuromuscular irAEs occurred within a median of 18 (range, 12–26) days after the most recent ICI dose and within a median of 18 (range, 12–108) days after the first ICI dose. The symptoms initially appeared after a median of one (range, 1–5) ICI cycle; there was a 3-week interval between the ICI cycles.

### GBS

The five participants with GBS had variable phenotypes, consisting of quadriparesis (*n* = 2) or paraparesis (*n* = 3) combined with hypesthesia; one showed sensory dominant symptoms, including sensory ataxia. Participant 1 required mechanical ventilator care, and participants 2, 3, and 4 required an assistive device for ambulation at the nadir. On electrodiagnostic testing, participant 3 had demyelinating features compatible with acute inflammatory demyelinating polyneuropathy (typical GBS), whereas participants 1 and 2 had axonal features compatible with acute motor and sensory axonal polyradiculoneuropathy (AMSAN subtype, GBS variant). Moreover, participants 4 and 5 had sensorimotor polyneuropathy with mixed axonal and demyelinating patterns. All four participants (participants 1, 2, 4, and 5) who underwent a cerebrospinal fluid (CSF) study had cytoalbuminological dissociation compatible with GBS, while the remaining participant (participant 3), who did not undergo a CSF study, had sensorimotor polyradiculoneuropathy indicative of GBS on electrodiagnostic testing. All three participants (participants 1, 2, and 5) who underwent spinal magnetic resonance imaging (MRI) exhibited cauda equina enhancement, compatible with GBS. Two of the five participants presented with thyroiditis as a concurrent systemic ICI toxicity.

### MG crisis with myositis

Four participants had quadriparesis, dyspnea, and ptosis, three of whom required mechanical ventilator support at the nadir; these participants had a previous history of ocular MG that was well controlled by symptomatic treatment, but they experienced MG crisis with newly developed myositis after ICI administration. The remaining participant (participant 9) had thymoma (type B3) without a history of MG but developed an MG crisis with myositis after ICI treatment. All four participants had elevated serum creatine kinase (CK) levels, suggestive of myositis (median: 2072 U/L, range: 527–7552 U/L, 2.2–56 times the upper limit normal), and positive results for anti-acetylcholine receptor antibodies at the time of MG crisis. All participants presented with concurrent hepatitis, and two of them also had myocarditis.

### Rescue treatment, clinical course, and outcomes

ICI treatment was discontinued and not re-administered to all nine participants. The median mRS score at the nadir was 4 (range, 3–5), indicating moderate-to-severe disability. The median duration from symptom onset to the initiation of rescue immunotherapy was 3 days (range, 1–20 days). All nine participants received corticosteroids as a part of the rescue treatment, which consisted of methylprednisolone (1 g daily) or prednisone (1–2 mg/kg daily), usually tapered by 10 mg prednisone each week. The median duration of corticosteroid therapy was 55.5 days (range, 10–75 days). Seven participants (five with GBS and two with MG crisis) also received intravenous immunoglobulin (IVIG, 2 g/kg for 2–5 days) concurrent with or subsequent to corticosteroid therapy, and two participants (one with GBS and one with MG crisis) additionally received plasmapheresis (5–6 cycles).

The median mRS score after 2 months of (usually completed) rescue treatment was 3 (range, 1–6). Participant 6, who had MG crisis with myocarditis, died due to rapidly progressive multi-organ failure despite the administration of rescue immunotherapy, which has been previously reported (Hyun et al. 2020). Of the eight survivors, one (with MG crisis) subsequently died of complicated pneumonia and two experienced subsequent cancer progression; however, the remaining five were stable. Three participants (two with MG crisis and one with GBS) required an assistive device for ambulation and long-term rehabilitation, whereas five could walk unassisted; three of whom had no significant disability after 2 months of rescue treatment.

## Discussion

Severe neuromuscular irAEs were rarely observed (0.6%) in this study, consistent with previously published results (less than 1%) (Cuzzubbo et al. 2017). ICI-induced neuromuscular irAEs were phenotypically heterogenous, which can be explained by the broad enhancing effects of ICIs on the priming and effector immune systems, which result in variable changes of immunological factors (Pardoll 2012; Ribas 2012). The majority (80%) of participants experienced neuromuscular irAEs after the initial ICI administration and within approximately 3 months of the initiation of ICI therapy, consistent with the previous reports, in which irAE onset occurred at a median of 6 weeks (approximately two cycles of ICIs) and within 3 months from the initiation of ICIs (Reynolds and Guidon 2019). All participants who experienced irAEs discontinued ICI therapy and were promptly administered corticosteroid rescue therapy after onset (within a median of three days), and their mRS scores generally improved. However, one participant with MG crisis and myocarditis died.

Taken together, our results suggest that ICI-induced severe neuromuscular irAEs are uncommon and that consequent neurological status usually improves with early rescue treatment. However, severe neuromuscular irAEs can be potentially life-threatening and result in long-term neurological sequelae. Clinicians should remain vigilant for irAEs once ICI therapy has been initiated, and should have a comprehensive understanding of the neuromuscular presentations of irAEs, so they can be recognized early and managed in a timely manner.

All five participants with GBS (variants) satisfied the Brighton criteria for the levels of diagnostic certainty (Fokke et al. 2014): participants 1 and 2 were level I, and participants 3, 4, and 5 were level II. Corticosteroid therapy is not generally recommended in idiopathic GBS (Hughes et al. 2016); but a trial of corticosteroid in ICI-induced GBS is recommended according to previously published guidelines (Brahmer et al. 2018; Thompson et al. 2019). IVIG was used concurrently with intravenous or oral corticosteroids as a rescue therapy, and all participants exhibited improved neurological status. In ICI-induced GBS, corticosteroid therapy may be worth considering as an active rescue treatment.

Concurrent MG crisis and myositis were usually accompanied by overlapping systemic irAEs such as hepatic dysfunction and/or myocarditis. Of the three participants who required mechanical ventilation in the intensive-care unit, one died, and two recovered, both with an mRS score of 4 (unable to attend to own bodily needs without assistance) after rescue therapy (Banks and Marotta 2007); all of them had a previous history of benign ocular MG. The remaining participant who had MG crisis with no history of clinical MG had an excellent recovery. Given that irAEs in participants with advanced thymoma presented with multi-organ involvement and could be potentially fatal, individualized risk stratification is essential before ICI treatment, particularly in these patients. Further investigations are needed to define the risk predictors (including a history of clinical MG) of neuromuscular irAEs.

This study was limited by potential unintentional bias as data were obtained from a single referral center; however, for the same reason, clinical characteristics and neuromuscular irAE outcomes could be consistently evaluated, and a relatively unified rescue therapeutic strategy was able to be provided to all participants (Brahmer et al. 2018; Thompson et al. 2019). Due to the small sample size and retrospective design, an optimal therapeutic regimen could not be suggested; therefore, future research is warranted to establish optimal management strategies.

## Data Availability

Anonymized data not described within this manuscript can be made available upon appropriate request from any qualified investigator.
